# SOX-1 antibodies in a patient with Crohn’s disease: a case report

**DOI:** 10.1186/s12883-022-02923-8

**Published:** 2022-11-02

**Authors:** Ennio Polilli, Antonella Frattari, Jessica Elisabetta Esposito, Gilda Angelini, Annalisa Di Risio, Elena Mazzotta, Simona Coladonato, Giancarlo Di Iorio, Giustino Parruti, Pierluigi Tocco

**Affiliations:** 1 Clinical Pathology Unit, Pescara General Hospital, Via Fonte Romana, 8, 65124 Pescara PE, Pescara, Italy; 2 Intensive Care Unit, Pescara General Hospital, Pescara, Italy; 3 Infectious Diseases Unit, Pescara General Hospital, Pescara, Italy; 4 Neurology and Stroke Unit, Pescara General Hospital, Pescara, Italy

**Keywords:** Anti-SOX1 antibodies, Crohn’s disease, Paraneoplastic neurological syndromes

## Abstract

**Background:**

The anti-SOX-1 antibodies have been mainly associated with Lambert-Eaton Myasthenic Syndrome (LETMS) and Small-Cell Lung Cancer (SCLC). In this report, we describe the interesting case of a patient with serum anti-SOX-1 antibodies and Crohn’s Disease (CD) with ensuing neurological symptoms.

**Case presentation:**

A Caucasian 67-year-old female was admitted to the Emergency Department with seizures, vertigo, emesis, nausea, postural instability and recurrent falls, over a period of 10 days. She had been affected by Crohn’s Disease since 1991. A CT scan failed to detect any ischemic or haemorrhagic lesion. A brain MRI revealed signs of leukoencephalopathy. Western blot analysis of her serum revealed a high titre of the onconeural antibody anti-SOX1, consistent with a neurological, cerebellar type, paraneoplastic syndrome. In spite of multiple efforts to unmask a possible underlying malignancy, no neoplastic lesion cropped up during hospitalization. Her clinical conditions progressively deteriorated, up to respiratory failure; a few days later she died, due to ensuing septic shock and Multiple Organ Failure.

**Conclusions:**

Our experience may usher and reveal a new role of anti-neural antibodies, so far reckoned an early indicator of associated malignancy, suggesting that neurological syndromes associated with such antibodies may complicate also chronic Gastrointestinal (GI) diseases. As of now, testing for anti-neuronal antibodies appeared unnecessary within the diagnostic assessment of gastroenterological disorders, which may lead to overlooking incident neurologic autoimmune diseases. Further exploration of such research hypothesis in clinical grounds appears intriguing.

## Background

Paraneoplastic Neurological Syndromes (PNS) are rare immune mediated neurological disorders caused by the immune cross-reactivity between antigens expressed by neurons and cancer cells [1, 2]. In these syndromes, anti-neuronal antibodies are considered diagnostic markers for both paraneoplastic neurologic disorders and the underlying cancer [3, 4]. Over the last decades, several onconeural autoantibodies, targeting intracellular antigens of neurons, have been reported. Among these, anti-SOX1 antibodies bind the SRY-Box Transcription Factor 1, a protein mainly expressed in the adult Bergmann glia of cerebellum, where it plays a key role in the development of the central nervous system (CNS) [5–7]. Relatively little is known about the mechanisms through which they trigger and constantly cause immune damage to neuron cells. However, many authors have pointed out that anti-SOX1 antibodies are oftentimes associated with a paraneoplastic form of Lambert-Eaton Myasthenic Syndrome (LEMS) and Small Cell Lung Cancer (SCLC) [6, 7]. Indeed, cancer has been detected in 93.5% of patients with anti-SOX1 antibodies, so that their presence is reckoned a powerful predictor of underlying SCLC [7]. Two types of PNS autoantibodies are described on the basis of the target antigen sites: intracellular, known as onconeuronal, and cell-surface autoantibodies. Several clinical features of paraneoplastic neurologic disorders are commonly found also in infective and degenerative disorders. PNS can affect central, peripheral or autonomic nervous system, being associated with paraneoplastic cerebellar degeneration (PCD), LEMS, encephalomyelitis, paraneoplastic limbic encephalitis (PLE), and sensory neuronopathy [7, 8].

Here we report on a patient who checked into the Emergency Department (ED) of Pescara General Hospital, with a history of chronic intestinal disorder and neurological symptoms. The patient had been suffering from Crohn’s Disease (CD) for approximately 20 years; she turned out positive for serum anti-SOX1 autoantibodies, in the absence of any underlying neoplastic disease.

## Case presentation

A 67-year-old Caucasian female was admitted to the ED of Pescara General Hospital on March 17th, 2021, for seizures, vertigo, emesis, nausea, postural instability, and recurrent falls, lasting approximately 10 days. She had been suffering from CD since 1991. In 2007, she underwent hemicolectomy with gastric resection and ileostomy.

The patient had a long history of Crohn’s Disease (CD), but she always declined biological therapies, repeatedly resorting on steroids to relieve her disease activity phases. During hospitalization due to refractory diarrhea, she was re-evaluated by a consultant gastroenterologist, who once more suggested to introduce steroids in therapy, considering her symptoms related to relapsing CD.

Neurological examination confirmed vertiginous syndrome with postural instability, difficulty in controlling eye movements with vertical nystagmus, and retropulsion. A brain CT scan did not detect any sign of ischemic or hemorrhagic lesions. At the time of hospitalization, the patient was on a treatment schedule with 500 mg/daily of mesalazine. The patient lost consciousness during her ED stay, being admitted into the Intensive Care Unit (ICU), where she was put on Propofol, Fentanyl and Rocuronium, in conjunction with intubation and mechanical ventilation. The next day her clinical status quickly improved; she was extubated and transferred to the Neurology unit, to complete her neurological diagnostic workup. An X-ray of shoulders reported a compound fracture of the humerus head, likely caused by one of her previous falls. A chest X-ray showed signs of bilateral pleural effusion. A brain MRI scan showed altered signal intensity in the peritrigonal region, corona radiata, and centrum semiovale of the brain, compatible with leukoencephalopathy. Cerebral Spinal Fluid (CSF) analysis showed no cells, negative Pandy’s reaction, glucose 60 mg/dL, and total CSF proteins 26 mg/dL. All viral and microbiological examinations carried out on CSF and serum turned out negative. Among immunological CSF examinations, normal levels of IgA (1.48, reference: 0.0-2.0 mg/L), IgG (10.3, reference: 4.8-58.6 mg/L) and IgM (0.417, reference: 0.0-2.0 mg/L) were reported. CSF and serum results of an electro-isofocusing analysis showed the presence of oligoclonal bands with mirror pattern (type 4), indicating the absence of intrathecal synthesis. Western blot analysis on serum revealed a high titre of onconeural anti-SOX1, compatible with a neurological, cerebellar type, paraneoplastic syndrome, whereas Amphiphysin-Ab, CV2-Ab, Ma2/Ta-Ab, Ri-Ab, Yo-Ab, Hu-Ab, Recoverin-Ab, Titin-Ab, zic4-Ab, GAD 65-Ab, Tr-Ab showed all negative results. Markers of cancer, including CEA (Carcinoembryonic Antigen), AFP (Alpha-fetoprotein), CA125 (Cancer Antigen 125), CA15.3 (Cancer Antigen 15.3), NSE (Neuron-Specific Enolase); markers of thyroid function (TSH, Thyroid-Stimulating Hormone; FT3, Free Triiodothyronine; FT4, Free Free thyroxine); antibodies against celiac disease; vitamin B12 and level of folic acid showed all normal values. Therefore, in the suspect of an autoimmune paraneoplastic encephalitis, an extensive search of a hidden tumour was started. A control brain MRI scan confirmed the presence of non-specific leukoencephalopathy, and a Total-body CT scan revealed only a suspected invagination of a jejunal loop, compatible with CD, whereas the presence of an underlying Small Cell Lung Carcinoma could be excluded, together with any lesion compatible with SCLC metastatic repetition.

In spite of therapy delivered (Immunoglobulin (Ig) therapy, 1 g/Kg/die for 5 days, cortisone therapy 20 mg twice/daily), her clinical conditions progressively worsened after approximately 10 days. A control chest X-ray documented left pleural effusion and hypodiafania, compatible with parenchymal inflammatory processes in the middle basal lobe. Sepsis and inflammatory biomarkers rose (PCT, Procalcitonin, 12.164 ng/mL, CRP, C-Reactive Protein, 133.71 mg/L), although blood cultures yielded persistently negative results. Severe thrombocytopenia (9000 PLT /uL) and anemia (7 g/mL Hemoglobin) ensued. After exclusion of Disseminated Intravascular Coagulation (DIC), the patient received high dose (0.5 g/Kg) Intravenous Immune Globulin infusion, as well as antimicrobial therapy associating 4.5 g piperacillin-tazobactam every 8 h; 50 mg tigecycline every 12 h and 70 mg/daily caspofungin. The patient desaturated to SO_2_ 85% and pO_2_/fO_2_ 256 mmHg. As a consequence, she was re-transferred to the ICU, where she died a few days later, due to refractory septic shock and refractory respiratory failure.

## Discussion and conclusions

In the case herein described, a yet unreported coexistence of CD and a neurologic disease triggered by an immune-mediated response due to anti-SOX1 autoantibodies is reported. CD is a heterogeneous Inflammatory Bowel Disease (IBD), affecting the gastrointestinal tract, with no in-depth or definite knowledge of its pathogenesis. Several researchers have hypothesised that tissue damage in IBD may be mediated by an abnormal immune response against the bacterial resident microflora in individuals with genetic susceptibility. Consequently, CD patients may be at risk of developing a second autoimmune disease, as in other autoimmune disorders [9–14]. Additionally, treatments for IBD, such as TNF inhibitors, have also been associated with an increased risk of new onset, overlapping autoimmune disorders [13, 15–19]. Therefore, several studies have identified an overlap status between IBD and various other immune-mediated diseases, such as ankylosing spondylitis, coeliac disease, psoriasis, systemic lupus erythematosus, rheumatoid arthritis, and multiple sclerosis. The most common extra-intestinal manifestations in CD involve joints, skin, eyes, coagulation system and liver [20, 21], whereas cerebral involvement was so far infrequently described in systematic reviews [22]. However, sporadic clinical cases were described, supporting the possible association between CD and polyneuropathy or vasculitis [23]. Polyneuropathy was reported in 0.9–3.6% of cases [24, 25], while very few cases of CD were found in association with posterior reversible encephalopathy syndrome [26], anterior ischemic optic neuropathy [27, 28] and myelopathy [29]. However, it is not yet clear whether the coexistence of these pathologies may represent a consequence of primary disease. All these findings considered, we found it worth sharing a clinical case with an uncommon overlap of a SOX1 related neurological syndromes and CD. Indeed, such association may turn out to be more frequent and relevant that reported so far. The majority of DPPX (potassium channel subunit dipeptidyl-peptidase-like protein-6) antibody-positive patients complain with Gastrointestinal (GI) symptoms, including constipation, abdominal pain, nausea, vomiting, early satiety, and diarrhea with weight loss [30], making the overlap of autoimmune neurologic and gastroenterological diseases an attractive field of research. Lutt et al. (2018) investigated the prevalence of IgG and IgA antineuronal antibodies in patients with gastrointestinal disease. Twenty-two of 44 patients with CD tested positive. However, they did not find any association with targeted antibodies, such as DPPX-Ab and Hu-Ab, determined by indirect immunofluorescence or by cell-based assays [30].

In our patient, anti-SOX1 antibodies were associated with neurological symptoms, most likely contributing to the severity of the clinical spectrum of the GI disease. However, as anti-SOX1 is a paraneoplastic antibody, associated with 93% of neoplastic disease cases, we also investigated the possible existence of an underling tumor, which could be consistently excluded. Our experience may therefore usher and reveal a new role of anti-neural antibodies, so far reckoned an early indicator of associated malignancy, suggesting that neurological syndromes associated with such autoantibodies may complicate also chronic GI diseases.

Onconeural antibodies are generally expressed within the immune response to an underlying tumour. Several described cases in the literature documented that their identification may precede cancer diagnosis [31]. Among onconeural antibodies, serum SOX-1 antibodies do not appear to be associated with a particular neurological phenotype [31], but were rarely described without the occurrence of an underlying tumour [32]. As a consequence, to exclude lung cancer, our patient underwent computed lung tomography (CT), considered a sensitive tool for the detection of lung nodules [31, 33]. As minute cancerous lesions may be missed in a chest examination by a CT [34, 35], when the initial radiological assessment does not detect any malignancy, most guidelines recommend [18F]-fluorodeoxyglucose positron emission tomography (FDG-PET) as the second-choice test, for its high sensitivity [31, 36, 37]. Clinical conditions of our patient, however, progressively worsened as described above, leaving us without a confirmatory diagnosis. Consent for an autopsy was asked, but it was unfortunately denied, as frequently reported for patients dying in neurological wards and in the ICUs [38].

We cannot exclude that gastrointestinal inflammation due to CD triggered by itself production of anti-SOX 1 antibodies [30]. Histopathological studies demonstrated that gut inflammation may induce several changes and abnormalities, including hypertrophy of ganglia and neuronal degeneration, in gut-associated neuronal cells [39, 40]. Interestingly, it has been known for over two decades that a member of SOX family protein as SOX-10 may be one of the earliest neural crest cell markers of Enteric Nervous System (ENS) progenitor cells [41]. In the adult gut of mammalians, they differentiate both in SOX-unexpressing neurons and in SOX-expressing glial cells. Experiments conducted in rodents suggested that enteric glial cells expressing SOX-10 protein may change their cellular program, to express a newly neurogenic potential [40]. Laranjeira et al. (2011) described that enteric SOX-10 expressing glial cells are capable of generating functional neurons in vivo after administration of an injury stimulus in the gut [40]. Taking into account all these findings, we may not exclude that production of autoimmune SOX antibodies was only related to relapsing gastrointestinal inflammation in CD. However, further studies are needed to clarify these intriguing aspects of our observation.

As of now, testing for antineuronal antibodies is not included in the diagnostic workup of gastroenterological disorders, which may lead, based on our and others’ pivotal reports, to overlooking incident neurologic autoimmune diseases in patients with CD. Further exploration of such research hypothesis in clinical grounds thus appears intriguing, possibly leading to a widening of our understanding of the disease spectrum of CD and to routine testing for antineuronal antibodies in gastroenterology wards.

## Data Availability

Data are available from the author upon reasonable request and with permission of Health Direction of Pescara General Hospital.

## Abbreviations

PNS: Paraneoplastic Neurological Syndromes
CNS: Central Nervous System
LEMS: Lambert-Eaton Myasthenic Syndrome
SCLC: Small Cell Lung Cancer
PCD: Paraneoplastic Cerebellar Degeneration
ED: Emergency Department
CD: Crohn’s Disease
ICU: Intensive Care Unit
MRI: Magnetic Resonance Imaging
CSF: Cerebral Spinal Fluid
DIC: Disseminated Intravascular Coagulation
IBD: Inflammatory Bowel Disease
GI: Gastrointestinal
CRP: C-Reactive Protein
CEA: Carcinoembryonic Antigen
AFP: Alpha-fetoprotein
Ca125: Cancer Antigen 125
Ca15.3: Cancer Antigen 15.3
NSE: Neuron-Specific Enolase
TSH: Thyroid-Stimulating Hormone
FT3: Free Triiodothyronine
FT4: Free thyroxine
PCT: Procalcitonin
ENS: Enteric Nervous System
FDG-PET: [18F]-Fluorodeoxyglucose Positron-Emission Tomography
CT: Computed Tomography
PLT: Platelet count
